# Structured Self-Rated Response to Iontophoresis with Verapamil and Dexamethasone in Peyronie's Disease

**DOI:** 10.1155/2014/957013

**Published:** 2014-04-03

**Authors:** Abas Kokab, Kevan Wylie, Patricia Allen, Abhijeeth Shetty, Debbie Davies-South

**Affiliations:** ^1^Sheffield Teaching Hospitals NHS Foundation Trust, Sheffield S10 2JF, UK; ^2^Sheffield Health and Social Care NHS Foundation Trust, Sheffield S11 9BF, UK

## Abstract

*Introduction*. New therapies evolve for the treatment of Peyronie's disease (PD) including the application of dexamethasone and verapamil using Electro Motive Drug Administration (EMDA). *Patients and Methods*. Patients with PD were routinely offered Potaba, Vitamin E, tamoxifen or colchicine for 6 to 18 months and for those with no improvement, 18 applications of dexamethasone and verapamil using EMDA occurred over a 6 week period. All 30 patients receiving EMDA therapy completed a questionnaire before and after treatment. The data was collected from December 2004 to November 2009 and analysed to evaluate the effectiveness of the treatment. *Results*. Median age of patients was 59 (range 39–71). Curvature was the most common presenting complaint (73.3%) followed by pain (23.3%), erectile dysfunction (13.3%), and lump (13.3%). 24/30 (80%) reported an improvement in symptoms after EMDA. 16 of the responders (66.7%) had a stable plaque for at least 6 months. The patients who complained of shortening of the penis (*P* = 0.003) or lowered sexual desire (*P* = 0.024) expressed subsequently significant response to treatment. There was statistically significant (*P* = 0.019) improvement of penile deviation reported by responding men. *Conclusion*. A significant proportion of patients who received EMDA reported decreased curvature following iontophoresis. No serious adverse reactions developed.

## 1. Introduction

Prevalence of Peyronie's disease (PD) is around 3–9% in males [1–3]. It is an inflammatory condition, which causes thickening of the tunica albuginea of the penis. PD is characterized by the formation of fibrous, noncompliant nodules within the tunica albuginea of the penis [4]. These fibrous plaques are not elastic; they impede the normal expansion of the penis and cause penile bending [5]. The most common site for a nodule to form is on the dorsal aspect of the penis; this is thought to be due to the structural arrangement of the tunica albuginea [6–8].

PD has a variable natural history ranging from a spontaneous regression in rare cases to progression with severe penile deformity [9, 10]. PD may lead to penile swelling, induration, shortening, and curvature in addition to painful erection and dyspareunia for both partners [11]. PD is associated with erectile dysfunction in more than 40% of cases [3, 12, 13]. The assessment and management of the disease have yet to be standardised. Treatment of PD presents several challenges to the clinician. There are a number of theories regarding the aetiology behind PD. Some pieces of evidence suggest that it may be the result of some repeated microvascular trauma or subtunical bleeding, through trauma to penis [11, 14–16]. The pathophysiology of PD seems to be as that of an exaggerated scarring process (Bjekic et al., 2006). However, its true pathogenesis remains unknown. As a result, the treatment of PD remains a problem and new therapies continue to evolve. The treatment of PD varies depending on its clinical manifestations and the current phase of the disease. The protocols to include medications have been applied to reverse, interrupt, or attenuate the underlying mechanisms with or without beneficial effects. Surgical procedures have been developed for patients who fail conservative measures and who have stable disease [17].

EMDA is a technique using a small electric charge to deliver a medicine through the skin transdermally. In patients with Peyronie's disease, transdermal iontophoretic administration of steroids [18] or orgotein [19] has shown promising results without the side effects associated with oral or intraplaque injection therapies. Two clinical studies using different methods showed that transdermal electromotive administration of verapamil and dexamethasone, with or without lidocaine, was beneficial in patients with Peyronie's disease [20, 21].

In this study, we analyse the response of patients with PD to iontophoresis with verapamil and dexamethasone as the second line of treatment.

## 2. Patients and Methods

A number of 208 patients with a diagnosis of PD were initially offered medications including Potaba, vitamin E, tamoxifen, or colchicine as per the local protocol for 6 to 18 months. Of these, for 30 men with no improvement and in the chronic phase of the PD, subsequent 18 applications of dexamethasone and verapamil using EMDA were carried out. This was over a 6-week period in Andrology clinic of Royal Hallamshire Hospital, Sheffield, UK. Peyronie's disease was diagnosed by a detailed medical and sexual history, a palpable plaque on physical examination, and, in many cases, photographic evidence or on ICI induced clinic erections.

The men were asked to complete a questionnaire [7] before and after treatment. The case notes of these 30 patients were retrospectively reviewed by the authors at this referral centre. The data was collected from December 2004 to November 2009 and analysed to evaluate the effectiveness of the treatment. Further to EMDA therapy the patients have been prescribed phosphodiesterase-5 (PDE-5) inhibitor pharmacotherapy for erectile dysfunction where required.

The treatment regime for each patient using the EMDA consisted of 3 treatments per week for six consecutive weeks (18 treatments in total). Before the treatment could begin, each patient underwent physical examination to establish suitability for this treatment modality. Before treatment could commence, the patient was asked to trim or shave the pubic hair at the base of the penis and also on the outer thigh to ensure good adherence and minimise leaks from the vessel. They were also asked to thoroughly degrease the skin above and around the plaque and on the thigh, using an alcohol swab. The patient was then asked to lie supine or gently elevated at the head before commencing each treatment. It was not possible for them to be sat upright, as this often resulted in further shortening of the penile shaft or protrusion of the lower abdomen making it difficult to keep the electrode vessel in place.

Once the patient was comfortable, the 5 mL plastic self-adhesive electrode vessel containing a silver-lined electrode (CT-DAS 500Ag, Physion srl, Medolla, Italy) was adhered to the penile skin overlying the plaque. The receptacle was then carefully filled with a solution of 4 mg/mL dexamethasone and 5 mg/mL verapamil (total 3 mL of solution). A dispersive electrode pad was then moistened with water and covered with conductive gel on one side. This was subsequently applied to the outer thigh ensuring total contact with the skin and gel and then secured in place with tape.

The anode (red plug) of the current generator (Physionizer 30, Physion srl, Medolla, Italy) was connected into the electrode vessel and the cathode (black plug) into the skin electrode pad on the thigh.

The physion box was then turned on; a pulsed direct current of 2.0 mA at 2 kHz was applied for 20 min. The time was automatically calculated by the physion box itself and an alarm sounded at the end of treatment.

Chi-square test, Fisher's exact test, and Mann-Whitney* U* test were used for statistical analysis. The level of significance was assumed 0.05.

## 3. Results

### 3.1. Age, Chief Complaint, and Medical History

The median age of the patients (*N* = 30) at presentation was 59 years (range 39–71). On average, men presented within 3.1 years of onset of their PD symptoms. Curvature was the most common presenting complaint among all patients (73.3%), followed by pain (23.3%), erectile dysfunction (13.3%), and lump (13.3%). All 30 patients who received iontophoresis with verapamil and dexamethasone had completed the questionnaires before and after the treatment. The clinical notes have also been reviewed on May 2010. 16 of these 30 (66.7%) patients had a stable plaque for at least 6 months. None of the cases had family history of Dupuytren's contracture, Lederhose disease, unusual scarring disorders, or severe straddle injury. None reported any serious side effects from the treatment.

### 3.2. Classification of Patients Based on Their Response to EMDA

24/30 (80%) reported an improvement in one or more symptoms, while 6/30 (20%) reported no benefit from EMDA therapy. The responder men (*N* = 24) reported an improvement in curvature (58.3%), pain (33.3%), lump (29.2%), or the quality of erection (20.8%) following treatment. This report of 30 consecutive patients presents a small group of nonresponders for comparison and the estimated power of statistical analysis is 0.833. When we stratified the cases according to the Kelami classification, among 30 patients 0 cases had demonstrated no curve; 19 cases described their curve as less than 30; 7 patients had curve of 31–60; and 4 of them had 60+. The nonresponder group included 2 patients with curvature of more than 60 as well as 3 patients with curvature between 31–60 and one case of curvature less than 30.

Age and duration of symptoms were not significantly different among two groups who either have responded or have shown no response to EMDA, as demonstrated in Table 1.

The results summarized in Table 1 indicate that the initial symptoms were not significantly different among two groups, except erectile dysfunction which was greater among the men who had no response to the treatment. The onset of deformity happened more suddenly among nonresponders, although this did not achieve significance. Shrinking of length of the penis was both more significantly prevalent and higher among men who responded to this treatment. The mean amount of shrinkage of length of the penis among responders was 2.29 cm but it was 0.33 cm in the nonresponder men; that is, if there was shrinking, the chance of response to this method of treatment was greater. Alteration to the sensation is not a good marker as to whether the man responded to EMDA therapy.

There was greater history of trauma to the penis either related to or without sexual activities in nonresponding men (Table 2). In the other words, men with trauma to their penises responded worse to the treatment. The patients suffered emotionally more than their partners, although the difference did not achieve significance.

The group of men who did not demonstrate twisting at arousal responded more to this method of treatment (Table 3). Another point is that twisting was more likely to happen at arousal rather than at rest in both groups (16.7 versus 25% in responder group and 0% versus 66.7% in nonresponder group). The course of curvature over the time was toward a worse situation among 75% of responders; however, it was in all nonresponder men.

Patients who expressed lowered sexual desire in the questionnaires completed before treatment more commonly demonstrated the benefit from EMDA therapy but the cases with high sexual desire responded less to the treatment (Table 4). The erectile firmness scored by patients prior to the treatment was not significantly different among responder and nonresponder men. While 37.5% of men who responded to EMDA scored their erection 10 out of 10, this figure was 66.7% among nonresponders. All nonresponders had complained of difficulty to penetrate before EMDA but just 45.9% of responders did. In both groups, the penetration problem was attributed by patients to be due to curvature rather than a lack of firmness and finally hinge effect. This suggests that if a man expresses difficulty with penetration, particularly due to curvature, he is less likely to benefit from EMDA. Rapid ejaculation was also more prevalent, frequent, and constant among responding men. In nonresponders, there was no recent complain of rapid ejaculation compared to 20.8% of responders.

In the medical history, some conditions such as alcoholism (twice), diabetes mellitus (three times), and tobacco smoking (almost three times) have been reported more frequently among the nonresponder group (Table 5). This is of interest as the latter two are considered risk factors for PD and may reflect a subgroup of this group of men. In the responder group, there was greater history of hypertension, hyperlipidemia, and vascular disease. Medications for erectile dysfunction were prescribed for all men in the nonresponder group; however, it was only in 33.3% of responding men. This difference achieved a significant difference (*P* = 0.005). A history of back trauma or surgery and current drinking habit was almost the same between two groups. Current tobacco smoking was more prevalent in the nonresponders (25.0% versus 66.7%).

The proportion of cases reporting improvement of curvature (58.35%), pain (33.30%), lump (29.2%), and erection (20.8%) is shown in Table 6. Statistically significant improvement of curvature was reported by men who responded to EMDA. Plaque was believed to be stable in 66.7% of responders and 50.0% of nonresponders. None reported any side effects from the treatment.

## 4. Discussion

The role of medical management in PD is controversial [22]. Medications such as potassium para-aminobenzozoate (Potaba) might prevent disease progression and improve pain; however, they do not reduce plaque size and do not cause disease regression clinically [22, 23]. Other medical agents such as vitamin E, tamoxifen, and colchicine have not demonstrated long term benefit to patients in placebo-controlled randomized trials [24–28]. Intralesional agents such as verapamil, steroid agents, and interferon have not been routinely adopted into clinical practice due to the limited evidence for their effectiveness [29–31]. Levine and Greenfield 2003, reported that 71.5% of excised tunica albuginea specimens from 14 men who received iontophoresis and topical verapamil therapy prior to undergoing surgical treatment for PD were found to contain measurable levels of verapamil [7]. A subsequent prospective, controlled study evaluated the efficacy of electromotive verapamil and dexamethasone compared to electromotive lidocaine in 96 men with PD [21]. Men were randomized to receive either verapamil and dexamethasone or lidocaine with a 2.4 mA electric current for 20 minutes, 4 times weekly for 6 weeks. Compared with base line, significant decreases in median plaque volume were seen in the actively treated group, whereas no changes in plaque volume or curvature were seen in the control group. Significant pain relief was experienced transiently in the control group and permanently in the treatment arm. These results support those of a previous uncontrolled study that reported plaque reduction in 82%, curvature decrease in 84%, and pain elimination in  88% of 49 men who received verapamil and dexamethasone treatment with iontophoresis [20]. The same group reported benefits although not statistically significant using verapamil versus saline in electromotive drug administration for PD in a double-blind, placebo-controlled trial involving 42 cases [32]. In this paper, we also report beneficial response to EMDA.

Age was not a factor to indicate the response to EMDA nor was duration of symptoms. The history of trauma to the penis during sex was more than twice in nonresponders (25% versus 67%). Injury to the penis that was not sex related was also more than twice as common in nonresponders (17.5% versus 50%). This may simply mean that those patients who had a traumatic PD are less likely to benefit from this treatment. Disease onset is commonly associated with preceding trauma and most often occurs in older men (mean age, 53 years; range, 19–83 years), although reports [33] have documented that majority of men with PD in their series had no specific recollection of trauma and 10% of patients experienced symptoms onset before 40 years of age.

Among both responders and nonresponders, the emotional impact of PD on patient was more prevalent and severe than the emotional impact on partners (patients with emotional suffering 85% versus partners 55%). However the emotional impact of PD is not fully addressed. It can be devastating to both the patient and his partner and may contribute to erectile difficulties. Men suffering from PD are at greater risk of depression, mood disturbances, low self-esteem, emotional distress, and relationship difficulties, and the quality of life of both the patient and partner may be significantly affected [1, 3, 34, 35]. Presence of PD might adversely influence the patient's virility and fear of partner sexual dissatisfaction [36]. Nelson et al. demonstrate that at least 48% of cases with PD suffer from mild or moderate depression [35]. The main cause of this psychological effect is the penile shortening [35, 37]. Further to treating the functional impairments of PD and treating penile symptoms, psychological impairment has to be addressed. Men should be supported and referred to mental health professionals if necessary.

Peyronie's disease is a condition with a diverse range of clinical manifestations. It may be that PD is not a single condition but rather an umbrella term for a number of distinct phenotypic anomalies that tend to produce deformity of the penis. The response to different methods of treatment could be different in one subset type of PD. For example, the current study demonstrated that the patients complaining from difficulty to penetrate are less likely to respond to EMDA (55.9% versus 100%). However rapid ejaculationwas reported three times higher by responders (16.7% versus 50%). This could be explained by the point that men who have coped with PD and have good sexual desire, firm erection, normal ejaculation, and active sex life may harbour overexpectation. This may be the reason of their negative responses in the self-reports questionnaires.

In the current study, the questionnaire [7] was not clear to some patients. The posttreatment questioner was the same as the pretreatment one. Many of our patients misunderstood the result of the last treatment, EMDA, with former methods of treatments. It also made our patients fill repetitive details in two similar forms. The suggestion is to design and validate another much simpler form to be used after the treatment.

## 5. Conclusion

A significant proportion of patients who received verapamil and dexamethasone by EMDA reported improvement of curvature. Patients most likely to benefit in our series were men reporting curvature <30 and shrinkage of the penis and where rapid onset and erectile dysfunction were not associated with the curvature. Patients who had traumatic PD or those complaining from difficulty to penetrate are less likely to benefit from this treatment. None reported any adverse effects.

## Figures and Tables

**Table 1:** Age at initial presentation, duration of symptoms, and clinical manifestations among responder and nonresponder men, expressed as mean (SD) for year and numbers (percent) for clinical manifestations.

Initial symptoms	Responder men	Nonresponder men	*P* value
Age; mean (SD)	57.96 (1.7)	59.00 (4.6)	0.705
Duration; mean (SD)	3.38 (0.3)	2.40 (0.3)	0.191
Curvature; *N* (%)	17 (70.8)	5 (83.3)	0.954
Sudden onset deformity; *N* (%)	11 (45.8)	4 (66.7)	0.651
Pain; *N* (%)	5 (20.8)	2 (33.3)	0.603
Lump; *N* (%)	3 (12.5)	1 (16.6)	0.481
ED; *N* (%)	2 (8.3)	2 (33.3)	0.169
Shortening; *N* (%)	23 (76.6)	2 (33.3)	**0.003**
Decreased sensation; *N* (%)	4 (29.2)	0 (0.0)	0.557
Numbness; *N* (%)	5 (20.8)	2 (33.3)	0.603
Painful sensation; *N* (%)	3 (12.5)	1 (16.7)	0.481

Bold refers to significant results.

**Table 2:** History of trauma to the penis and emotional impact following Peyronie's disease among responder and nonresponder men and their partners, expressed as percentage.

	Responders (%)	Nonresponders (%)	*P* value
Trauma to penis during sex	25	66.7	0.141
Injury to penis not sex related	17.5	50.0	0.110
Emotional impact on patient	91.7	83.3	0.501
Emotional impact on partner	66.7	50.0	0.641

**Table 3:** Penile deformities including curvature, twisting, and other deformities among responder and nonresponder men, expressed as percentage.

Penile deformity	Responders (%)	Nonresponders (%)	*P* value
Curvature, up	79.2	83.3	1.0
Curvature, down	8.3	0.0	1.0
Curvature, left	12.5	16.7	0.481
Worsen curvature	75.0	100.0	0.302
Twisting, at rest	16.7	0.0	0.557
Twisting, aroused	25.0	66.7	0.141
Other deformity	45.8	16.7	0.358

**Table 4:** Self-report on quality of sex and erectile and sexual functions prior to EMDA therapy among men who either expressed benefit or not improved using the treatment.

Sexual functions	Responders (%)	Nonresponders (%)	*P* value
Capable to sexual intercourse	50.0	83.0	0.196
Able to ejaculate	87.5	100.0	1.0
Rapid ejaculation	50.0	16.7	0.196
Painful sex for partner	25.0	50.0	0.329
Difficulty with penetration	45.9	100.0	0.061
Due to curvature	25.0	66.6	0.141
Due to hinge effect	4.2	16.5	0.366
Due to lack of firmness	16.7	16.9	1.0
Normal sexual desire	45.8	66.7	0.651
High sexual desire	4.2	33.3	0.094
Low sexual desire	50.0	0.0	**0.024**
Erectile score (0–10), mean ± SD	7.6 ± 0.7	7.7 ± 0.8	0.754
Cases with 10/10	37.5	66.7	0.360
Maintain erection after penetration	66.7	50	0.641
Morning erection	54.2	50	1.0

Bold refers to significant results.

**Table 5:** Medical history of men either responded or not improved using EMDA.

Past Medical History	Responders (%)	Nonresponders (%)	*P* value
Alcoholism	8.3	16.7	0.501
Medications for ED	33.3	100	**0.005**
Diabetes mellitus	4.7	33.3	0.128
High blood pressure	9.5	0.0	0.064
Elevated cholesterol	38.0	16.7	0.633
Coronary heart disease	14.3	16.7	1.00
Back trauma or surgery	19.0	16.7	—
Other vascular disease	52.4	33.3	0.986
Current tobacco smoker	25.0	66.7	0.141
Current drinker	70.8	83.3	0.358

Bold refers to significant results.

**Table 6:** Beneficial changes reported by patients following EMDA.

Improvement	Responders, *N* (%)	Nonresponders, *N* (%)	*P* value
Improved curvature	14 (58.3)	0 (0.0)	**0.019**
Stable plaque	16 (66.7)	3 (50.0)	0.641
Less pain	8 (33.3)	0 (0.0)	0.155
Improved lump	7 (29.2)	0 (0.0)	0.290
Improved erection	5 (20.8)	0 (0.0)	0.553

Bold refers to significant results.
